# Regulation of the tuft cell-ILC2 circuit in intestinal mucosal immunity

**DOI:** 10.3389/fimmu.2025.1568062

**Published:** 2025-04-28

**Authors:** Kaiyu Shang, Xinxin Qi, Tingting Tian, Huidong Shi, Yuejie Zhu, Fengbo Zhang

**Affiliations:** ^1^ Department of Clinical Laboratory, The First Affiliated Hospital of Xinjiang Medical University, Urumqi, Xinjiang, China; ^2^ Reproductive Medicine Center, The First Affiliated Hospital of Xinjiang Medical University, Urumqi, Xinjiang, China

**Keywords:** tuft cells, mucosal immunity, intestinal homeostasis, inflammatory bowel disease, tuft cell-ILC2 circuit

## Abstract

The intestinal mucosal immune system maintains homeostasis through complex interactions between epithelial cells and innate lymphoid cells in the lamina propria. Tuft cells, previously overlooked intestinal epithelial cell types, detect parasites and metabolites via Sucnr1 and TAS2R receptors. They secrete IL-25, which activates type 2 innate lymphoid cell (ILC2) via the IL-25R receptor. ILC2 releases IL-13, resulting in further promotion of tuft and goblet cells from stem cells. This positive feedback loop amplifies the local type 2 immune response, combating parasitic infections. Tuft cells also recognize viruses and bacteria, but the role played by the tuft cell-ILC2 circuit in this process is not yet clear. Furthermore, tuft cell-ILC2 circuit is influenced by dietary fiber, intestinal microbiota, and other factors, contributing to new functions in maintaining intestinal homeostasis. In inflammatory bowel disease, this immunological circuit may be protective. This review summarizes the current understanding of the tuft cell-ILC2 circuit, its regulatory mechanisms, and potential implications in intestinal disease. Graphical abstract (by Figdraw 2.0)

## Graphical Abstract

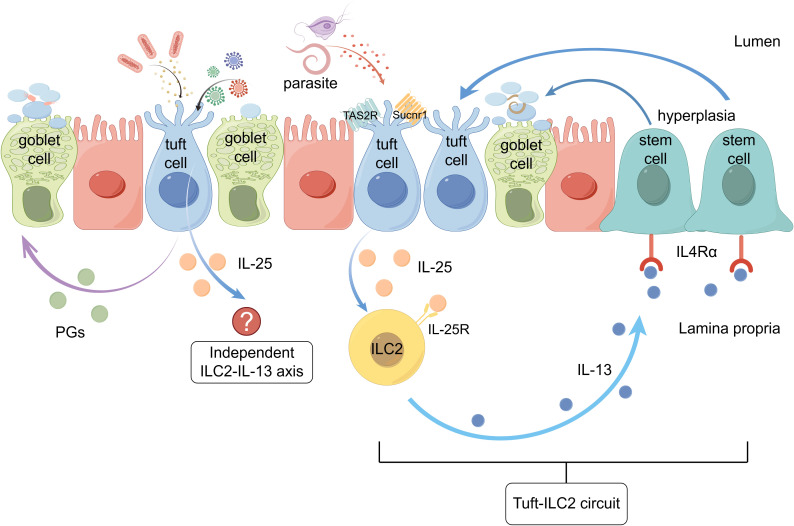

## 1 Introduction

The intestinal mucosa functions as a crucial interface between the host and the external environment, continually exposed to a diverse dietary antigens, commensal microorganisms, and potential pathogens. To maintain homeostasis within this complex ecosystem, the mucosal immune system has developed sophisticated mechanisms that allow it to tolerate innocuous substances while mounting protective responses against threats such as helminths, protozoa, and pathogenic bacteria (1). Among the various immune responses coordinated within the intestinal mucosa, type 2 immunity serves a pivotal role in both protection against parasites and tissue repair. Type 2 immune responses are characterized by the production of specific cytokines, including interleukin (IL)-4, IL-5, IL-9, and IL-13, and involve intricate interactions between adaptive and innate immune cells, such as type 2 helper T cells 2 (Th2), type 2 innate lymphoid cell (ILC2), eosinophils, mast cells, basophils, and alternatively activated macrophages (2, 3).

Intestinal epithelial cells (IECs) have emerged as critical mediators in the initiation and regulation of type 2 mucosal immunity. Among these, tuft cells, a rare epithelial cell population, have recently been identified as essential sentinels that detect parasitic infections and orchestrating downstream type 2 responses (4–6). Named for their distinctive apical microvilli protrusions, tuft cells were first described in the rat trachea by Rhodin and Dalhamn in 1956 (7). Although subsequently identified in various mucosal tissues, including the intestine, their functional significance remained largely unexplored for decades. In 2016, three independent research groups demonstrated that intestinal tuft cells function as the primary source of IL-25, a crucial initiating cytokine for type 2 immunity (4–6). When tuft cells sense parasites or their metabolites, they secrete IL-25, which activates ILC2 to produce IL-13. IL-13 subsequently acts on undifferentiated epithelial progenitor cells, promoting the differentiation and amplification of tuft cells and goblet cells, thereby enhancing mucus secretion and facilitating helminth expulsion (4, 8). This positive feedback mechanism is referred to as the “tuft cell-ILC2 circuit” (9). The tuft cell-ILC2 circuit has been demonstrated to elicit the classic “weeping and sweeping” response against various helminth parasites. This protective reaction (4–6) is remarkably conserved across different mouse strains and operates independently of adaptive immunity (4, 5).

Recent studies have further illuminated the profound impact of the tuft-ILC2 circuit on intestinal physiology and homeostasis. Activation of this circuit drives small intestinal remodeling, including villus elongation, crypt hypertrophy, and intestinal smooth muscle hypercontraction (10, 11). At the molecular level, advances in single-cell genomics and genetic manipulation techniques have provided unprecedented insights into the signaling pathways and transcriptional networks governing tuft cell development and function. For instance, tuft cells express canonical taste transduction proteins, highlighting their specialized role in chemosensation (12, 13).

Despite significant advances in understanding this field, numerous questions remain regarding the regulation and function of the tuft cell-ILC2 circuit in both health and disease. Specifically, the precise molecular mechanisms by which tuft cells detect and respond to various stimuli, the potential heterogeneity and plasticity among tuft cell subpopulations, and the long-term consequences of tuft cell activation on intestinal homeostasis and systemic immunity require further investigation. In this review, we synthesize current knowledge about the tuft cell-ILC2 circuit in intestinal type 2 mucosal immunity, highlight recent mechanistic discoveries, and discuss its implications for host defense, physiology, and pathology. It is important to emphasize that the majority of findings on tuft cell biology and the tuft cell-ILC2 circuit described in this review are derived from mouse models, with comparatively limited data currently available from human studies.

## 2 Tuft cells: sentinels of the intestinal epithelium

### 2.1 Overview of tuft cells

**Figure 1:**
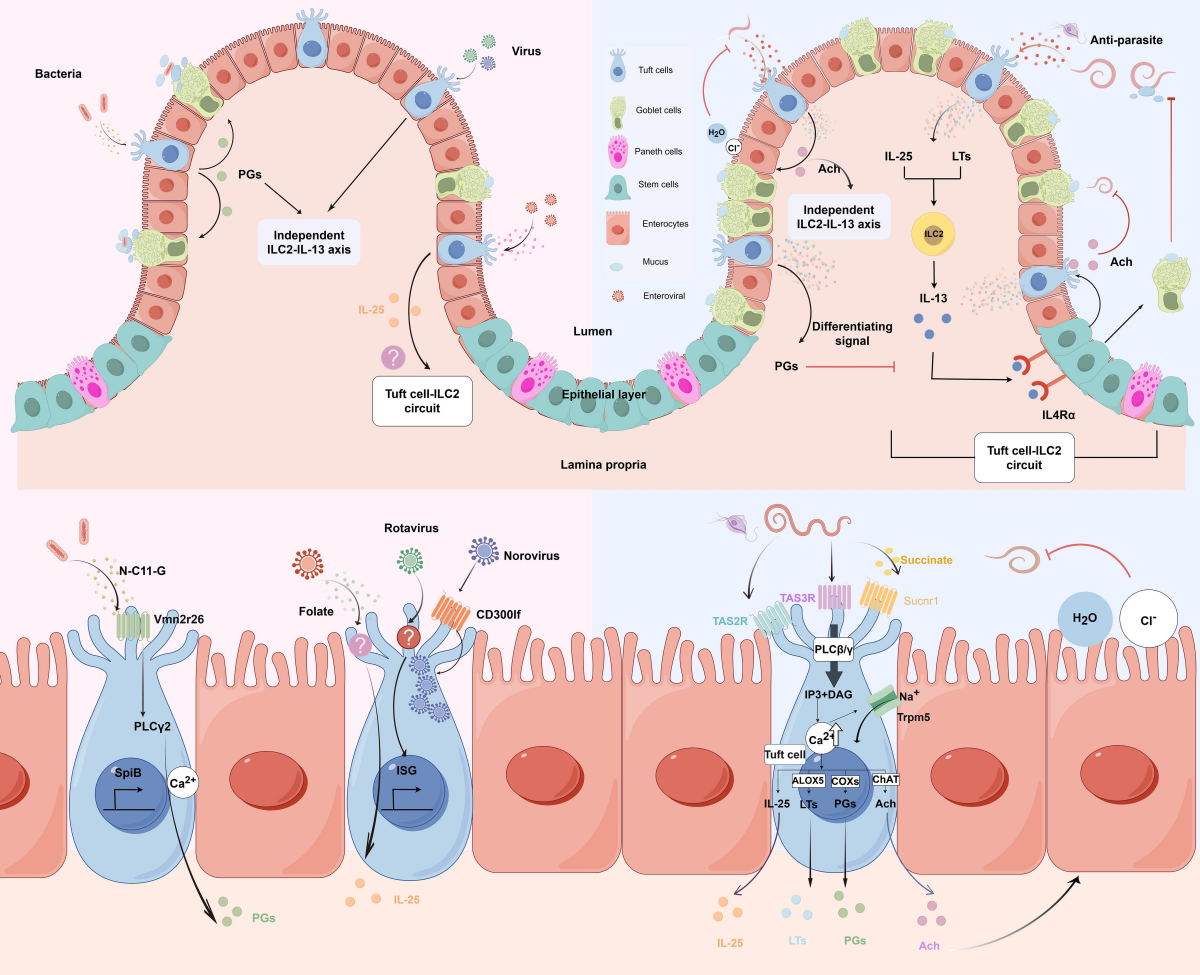
Molecular and cellular signaling of intestinal tuft cells in response to infections. (by Figdraw 2.0). Left panels: Tuft cell detection of bacterial and viral infections. Upper left: Overview of tuft cells recognizing bacterial and viral pathogens. Lower left: Magnified view of molecular mechanisms. Bacterial metabolite N-C11-G from *Shigella* is recognized by Vmn2r26 GPCR expressed on tuft cells, activating PLCγ2-Ca^2+^ signaling pathway. This leads to PGs production and SpiB-induced tuft cell hyperplasia. Norovirus binds to CD300lf receptors on tuft cells, leading to viral replication. Rotavirus triggers tuft cell activation through an unknown receptor, resulting in ISG expression that contributes to antiviral defense. Enteroviruses upregulate IL-25 expression and induce tuft cell expansion through folate metabolism, but it is uncertain whether it activates type 2 immune responses. Right panels: Tuft cell-mediated response to parasitic infections. Upper right: Overview of tuft cells in anti-parasitic immunity. Tuft cells detect parasite metabolites and produce IL-25 and LTs, which activate ILC2 to secrete IL-13. IL-13 binds to IL4Rα on intestinal stem cells, promoting differentiation into tuft cells and goblet cells. Goblet cells secrete mucus to aid in parasite clearance, establishing the tuft cell-ILC2 circuit. However, PGs counteract IL-13 effects. Moreover, tuft cells secrete Ach which can directly inhibit helminth reproduction or act on adjacent epithelial cells, inducing chloride secretion followed by water secretion, thereby promoting worm expulsion. Lower right: Magnified view of molecular mechanisms. Succinate binds to Sucnr1, while TAS2R and TAS3R, all GPCRs, detect other parasite-derived metabolites. Receptor activation triggers phospholipase C (PLCβ/γ), converting PIP2 to IP3 and DAG, leading to increased intracellular Ca^2+^ and Trpm5 channel opening. This cascade results in IL-25 release, enhanced ChAT expression, and activation of eicosanoid pathway-related proteins (COX1, COX2, and ALOX5). Adapted from Seminars in Cell & Developmental Biology, Vol. 150-151, Bas J, Jay P, Gerbe F, Intestinal tuft cells: Sentinels, what else?, Pages 35-42, Copyright (2023), with permission from Elsevier.

Tuft cells are a specialized type of epithelial cell found in various organs, including the olfactory epithelium, taste buds gallbladder, and the luminal gastrointestinal tract (14). Notably, tuft cells are absent in the lungs and pancreatic ducts under normal physiological conditions but can emerge during inflammatory processes, tissue injury, or disease states (15, 16). Tuft cells are relatively rare, comprising only 0.4% of the total intestinal epithelial cell population in adult mice under steady-state conditions, and they are distributed fairly evenly from the duodenum to the colon (5). Morphologically, tuft cells exhibit a distinctive bottle-shaped, characterized by a narrow base and a wider apical pole. Their signature feature—clusters of microvilli that extend into the lumen—is the basis for their designation as ‘tuft’ cells (12, 17). Ultrastructurally, each apical microvillus is supported by a bundle of axial actin-like filaments that extend deep into the cell body, forming extraordinarily long rootlets. In addition to the apical microvilli, tuft cells also possess a second set of laterally-projecting microvilli that extend from the cell surface into invaginations of adjacent epithelial cells (18).(**Figure 1**) Tuft cells are further distinguished by the presence of a unique organelle known as the tubuloveolar system, which consists of vesicles and tubular structures located in the supranuclear region (19). In addition, Haber et al. utilized single-cell RNA sequencing (scRNA-seq) to reveal the heterogeneity within the tuft cell population, categorizing intestinal tuft cells into two distinct subpopulations: Tuft-1 and Tuft-2, based on their gene expression profiles. Tuft-1 cells are characterized by elevated expression of genes associated with neuronal development, whereas Tuft-2 cells exhibit enhanced expression of immune response-related genes, including type 2 cytokine receptors such as IL4ra (IL-4), IL13ra1 (IL-13), and IL17rb (IL-25) (20). However, more recent spatial transcriptomic analysis by Manco et al. revealed that these tuft cell subpopulations may actually represent different maturation stages along the crypt-villus axis rather than distinct lineages, with tuft-1 cells being more abundant at the villus bottom while tuft-2 cells predominantly localized at the villus tip (21). This spatial patterning suggests a continuous differentiation process as tuft cells migrate along the villus axis, rather than separate developmental trajectories.

In essence, tuft cells constitute a rare but distinctive epithelial population characterized by their bottle-shaped morphology, specialized microvilli arrangements, and unique tubuloveolar system. Recent transcriptomic analyses reveal their spatial heterogeneity along the crypt-villus axis.

### 2.2 Molecular markers in tuft cells

Tuft cells express a variety of molecular markers, each associated with specific cellular functions. The earliest molecular markers used to identify tuft cells were linked to their unique ultrastructural characteristics (17). Villin and Fimbrin are enriched in the apical membrane of tuft cells and play roles in actin binding and microfilament bundle formation. However, their utility as specific markers is limited due to cross-reactivity with other epithelial cell types (18). Doublecortin-like kinase 1 (DCLK1) is highly expressed in most differentiated tuft cells (22), with the exception of those in taste buds and olfactory epithelium, and participates in microtubule polymerization, making it a widely used tuft cell marker (22). Notably, while DCLK1 serves as a reliable marker in murine models, it is not expressed in human tuft cells (23). Acetylated α-tubulin, found in the apical membrane, protects long-lived microtubules from mechanical stress damage (24). Additionally, cytokeratin 18 shows stronger expression in tuft cells compared to other intestinal epithelial cells, contributing to maintenance of cellular structural integrity. Ankyrin is located in the basolateral membrane of tuft cells, contributing to cell mechanical stability. However, due to the substantial expression of ankyrin in the basolateral membrane of intestinal epithelial cells generally, tuft cells cannot be easily distinguished using ankyrin antibodies alone (25). In addition, tuft cells, functioning as chemosensory cells, express a variety of key proteins involved in taste signal transduction, including α-gustducin, phospholipase Cβ2 (PLCβ2), transient receptor potential cation channel subfamily M member 5 (Trpm5), choline acetyltransferase (ChAT), and taste receptor type 1 member 1 (T1R1) and taste receptor type 1 member 3 (T1R3) (26). α-gustducin is a G protein α subunit that couples to taste receptors upon activation, triggering an intracellular signaling cascade. PLCβ2 hydrolyzes phosphatidylinositol 4,5-bisphosphate (PIP2) to generate the second messenger inositol 1,4,5-trisphosphate (IP3) and diacylglycerol (DAG), which mediate an increase in intracellular calcium ion concentration. Trpm5, a transient receptor potential cation channel highly expressed in taste cells (27), is activated by intracellular calcium ions, leading to cell depolarization and ultimately triggering action potentials (28). Tuft cells are unique among epithelial cells in that they express ChAT (29), which catalyzes the synthesis of acetylcholine (ACh). Research by Ndjim et al. indicates that when the host is infected with worms, the secretion of ACh in intestinal tuft cells increases, and ACh inhibits the reproductive ability of worms through interaction with worm muscarinic receptors (30). Recent findings by Billipp et al. demonstrate another critical function of tuft cell-derived ACh during helminth infection. It promotes epithelial chloride secretion in the small intestine, which enhances water secretion and contributes to intestinal helminth clearance, independent of its effects on type 2 inflammation (31). T1R1 and T1R3, members of the taste receptor family, are present in most tuft cells and may function in amino acid perception (32).

In addition to structural and chemosensory markers, tuft cells express several other important molecules. Firstly, tuft cells specifically express the transcription factor *Pou2f3*, which is critical for lineage commitment (33, 34). Research conducted by Gerbe and colleagues has demonstrated that *Pou2f3* is indispensable for the formation and maintenance of tuft cells, and its deletion results in the complete absence of tuft cells (5). Furthermore, the zinc finger transcriptional repressor GFI1B is also highly expressed in tuft cells. GFI1B is known to be related to GFI1, which stabilizes goblet cells and Paneth cells by inhibiting the pro-endocrine *Neurog3* gene (13). However, the specific function of GFI1B in tuft cells remains to be further elucidated.

Secondly, tuft cells express cyclooxygenase 1 (COX1), cyclooxygenase 2 (COX2) (12) and hematopoietic prostaglandin D2 synthase (H-PGDS), which are associated with prostaglandin synthesis. Prostaglandins are a crucial class of lipid mediators involved in various physiological processes, including inflammation, immune regulation, and vasomotion. COX1 and COX2 serve as key enzymes in the prostaglandin synthesis pathway, while H-PGDS specifically facilitates the production of prostaglandin D2 (PGD2). During intestinal helminth infection, immune cells—particularly ILC2—secrete abundant interleukin-13 (IL-13). IL-13 binds to the IL-13 receptor α1 subunit (IL-13Rα1) on the surface of intestinal stem cells (ISCs), triggering a series of cellular changes: epithelial cell proliferation is inhibited, terminal differentiation is accelerated, Lgr5-expressing stem cells are depleted, and the quantities of goblet cells and tuft cells increase while the expression of the PGD2 receptor, chemoattractant receptor-homologous molecule expressed on TH2 cells (CRTH2), is significantly upregulated. The expansion of goblet cells leads to increased mucus production, which helps to expel the helminths. Moreover, the expanded population of tuft cells secretes more PGD2, which acts on CRTH2-expressing ISCs, counteracting the effects of IL-13 by promoting epithelial cell proliferation, suppressing excessive goblet cell accumulation, and downregulating IL-13Rα1 expression levels. In summary, during helminth infection, the PGD2-CRTH2 pathway prevents excessive inflammation and tissue damage while still allowing for efficient anti-helminth effector mechanisms (35).

In addition, tuft cells secrete the type 2 cytokine IL-25, which can directly activate type 2 innate lymphoid cell (ILC2) in the intestinal lamina propria to produce IL-13. This cytokine, IL-13, subsequently acts on undifferentiated progenitor cells in the epithelial crypts, promoting their differentiation into tuft cells and goblet cells, thereby mediating immune responses to parasitic infections. Under steady-state conditions, IL-25 derived from tuft cells maintains the levels of IL-13 secreted by ILC2. Following intestinal infection by worms, the number of intestinal tuft cells increases significantly, and goblet cells also experience hyperplasia. In mice with a specific knockout of intestinal epithelial IL-25, the proliferative responses of tuft cells and goblet cells induced by parasite infection were significantly diminished. These findings suggest that tuft cell-derived IL-25 plays a crucial regulatory role in intestinal anti-helminth immunity (4). Furthermore, Schneider et al. discovered that the G protein-coupled receptor Sucnr1, expressed by tuft cells, can detect the metabolite succinic acid produced by pathogens (10, 36). In addition to helminths, several other microorganisms, such as bacteria, yeasts, and fungi, also produce succinic acid. However, it remains unclear whether the succinic acid produced by these diverse pathogens is universally detected by the Sucnr1 receptor in tuft cells, and if so, whether it elicits similar or distinct immune responses. Different types of pathogens often trigger specific patterns of immune responses. For instance, bacteria and fungi predominantly activate type 1 immunity, characterized by the production of pro-inflammatory cytokines and the recruitment of neutrophils and macrophages. The extent to which tuft cells discriminate between the succinic acid signals derived from various pathogens and orchestrate the appropriate type of immune response warrants further investigation.

Collectively, tuft cells express diverse molecular markers associated with their structural, chemosensory, and immune functions. These include cytoskeletal proteins (villin, fimbrin, DCLK1, acetylated α-tubulin), taste transduction molecules (α-gustducin, PLCβ2, Trpm5, T1R1/T1R3), and transcription factors essential for lineage commitment (*Pou2f3*, GFI1B). Functionally critical markers include ChAT for acetylcholine synthesis, COX1/COX2 for prostaglandin production, and IL-25 for immune activation. Sucnr1 receptor enables detection of microbial metabolites.

### 2.3 Tuft cells are initiators of type 2 mucosal immunity in the intestine

#### 2.3.1 Tuft cells can sense a variety of helminths and protists

Current research has demonstrated that the intestinal infection of mice with various helminths, such as *Nippostrongylus brasiliensis* (4, 5), *Heligmosomoides polygyrus* (5), and *Trichinella* sp*iralis* (26), as well as protists like *Tritrichomonas* (10), can lead to a significant increase in the number of tuft cells. This raises important questions about how tuft cells detect these pathogens and what effector functions they perform during infection.

Studies indicate that tuft cells can sense pathogens and their metabolites through specific receptors. Most of these receptors belong to the G protein-coupled receptor (GPCR) family, including the succinate receptor 1 (Sucnr1) and the bitter taste receptor (TAS2R). The primary ligand currently identified for tuft cells is succinate, a metabolite produced by helminths. Succinate binds to the Sucnr1 on the surface of tuft cells, leading to an increase in intracellular Ca^2+^ concentration, which subsequently triggers the opening of the cation channel Trpm5. The opening of the Trpm5 channel initiates a cascade of downstream reactions, culminating in multiple effector functions (6, 10, 37).

Research by Nadjsombati and colleagues indicates that although both the worm *Nippostrongylus brasiliensis* and the protist *Tritrichomonas rainier* secrete succinic acid and elicit type 2 immune responses, the sensing mechanisms of tuft cells for these two pathogens may differ. Specifically, the immune response of tuft cells to *Nippostrongylus brasiliensis* is largely independent of Sucnr1, while the response to *Tritrichomonas rainier* is entirely reliant on Sucnr1. This distinction suggests that, although Sucnr1 plays a role in the detection of certain pathogens by tuft cells, there may also exist Sucnr1-independent sensing pathways for different pathogens (37).

Recent studies by Billipp et al. (31) and Ndjim et al. (30) have revealed that tuft cells play dual roles in response to helminth infections — as both sentinel and effector cells. As sentinel cells, they initiate type 2 immune responses by releasing IL-25, which activates ILC2. Once activated, ILC2 releases type 2 cytokines, particularly IL-13, which targets intestinal epithelial cells via the IL4Rα-dependent pathway. This leads to the reprogramming of epithelial progenitor cells, resulting in the substantial expansion of goblet cells and tuft cells (5). The hyperplastic goblet cells produce large quantities of mucus, which assist in the elimination of worms (38). Importantly, these studies have uncovered a critical effector function of tuft cells: the production and release of ACh. Tuft cells are the only intestinal epithelial cells that express ChAT, the enzyme responsible for ACh synthesis. During helminth infection and the resulting type 2 immune response, tuft cell hyperplasia is accompanied by increased ACh production per tuft cell. This ACh is released into the intestinal lumen, where it directly impacts helminth physiology by targeting parasite muscarinic ACh receptors. Ndjim et al. (30) demonstrated that ACh exposure significantly reduces helminth fecundity, providing a novel mechanism by which tuft cells directly contribute to anti-parasitic defense. Billipp et al. (31) further showed that tuft cell-derived ACh also acts on neighboring epithelial cells to induce secretion of chloride and subsequently water, contributing to the “weep” component of the “weep and sweep” response that facilitates worm expulsion.(**Figure 1**)

In addition to ACh and IL-25, tuft cells also produce lipid mediators through the activation of eicosanoid pathway-related proteins, including cyclooxygenases (COX1, COX2) and arachidonic 5-lipooxygenase (ALOX5) (12, 39). These enzymes facilitate the production of PGs and LTs, particularly leukotriene C_4_ (LTC_4_), which can also activate ILC2 and contribute to the type 2 immune response. The newly generated tuft cells from this process further amplify the type 2 immune response, creating a positive feedback circuit. This dual function of tuft cells — as both initiators of type 2 immunity through IL-25 secretion and as direct effectors through ACh release — highlights their central role in coordinating anti-helminth immunity in the intestinal epithelium. (**Figure 1**)Fundamentally, tuft cells recognize helminth and protist metabolites through specialized receptors, triggering calcium-dependent signaling cascades that culminate in immune mediator release. Their dual functionality—initiating type 2 immunity via IL-25 while directly targeting parasites through acetylcholine secretion—establishes them as both sentinel and effector cells in anti-helminth defense.

#### 2.3.2 Role of tuft cells in bacterial and viral infections

Tuft cells play a crucial role in sensing and responding to intestinal bacterial and viral pathogens. For instance, the bacterial metabolite N-undecanoylglycine (N-C11-G), produced by *Shigella*, can be detected by the Vmn2r26 GPCR expressed on tuft cells. This recognition activates the PLCγ2-Ca^2+^ signaling pathways within tuft cells, resulting in increased production of prostaglandin D2 (PGD2), which enhances mucus secretion from goblet cells. The thickened mucus layer effectively separates bacteria from the intestinal epithelium and facilitates their elimination through the “flushing” mechanism. Unlike the anti-parasitic response which depends on the tuft cell-ILC2-IL-13 circuit, this antibacterial function operates independently of type 2 immune responses. Furthermore, the bacterial sensing promotes tuft cell expansion through increased expression of Spi-B transcription factor (SpiB), creating a positive feedback circuit that amplifies the protective response. This mechanism effectively prevents bacterial adhesion to the epithelium and subsequent invasion (40). (**Figure 1**)

In addition to their role in sensing bacteria, tuft cells are also implicated in viral infections. Norovirus, a prevalent cause of gastroenteritis, has been shown to specifically bind to the Cd300lf receptor on mouse tuft cells, facilitating its infection and replication within these cells. Mice treated with the cytokines IL-4 and IL-25, which promote type 2 immune responses, exhibited a significant increase in the number of tuft cells in their intestines. However, these mice also demonstrated heightened susceptibility to infection with mouse norovirus (MNoV). This observation suggests that while type 2 immunity typically aids in the clearance of pathogenic infections, an excessive type 2 immune response may instead enhance viral replication in tuft cells during norovirus infection (41). Nonetheless, current studies have not identified CD300lf expressed by human tuft cells as a receptor for human norovirus, indicating that tuft cells may serve as norovirus host cells primarily in rodent models (42). (**Figure 1**)

Recent groundbreaking research by Strine et al. (43) revealed that tuft cells create an immune-privileged niche that enables norovirus persistence by evading adaptive immunity. This occurs because tuft cells express minimal MHC class I and lack MHC class II molecules, which prevents effective antigen presentation to T cells. Despite inducing functional norovirus-specific CD8^+^ T cells, this immune evasion strategy allows norovirus to establish persistent infection within tuft cells. Moreover, Strine et al. (44) identified both tuft-cell-intrinsic factors (such as viral receptor expression) and tuft-cell-extrinsic factors (such as immunomodulatory signaling from non-epithelial cells) that collectively determine norovirus tropism and regulate viral immunity.

The complex interplay between interferons and tuft cells represents a critical bottleneck for norovirus persistence. Ingle et al. (45) demonstrated that interferon-λ (IFN-λ) derived from nonsusceptible enterocytes acts directly on tuft cells to limit persistent norovirus infection. This discovery highlights the importance of intercellular communication in antiviral defense, where virus-sensing by non-infected cells (enterocytes) generates antiviral signals that protect susceptible cells (tuft cells). Moreover, using innovative barcoded virus strategies, Aggarwal et al. (46) demonstrated that IL-4-induced tuft cell expansion increased both viral diversity and viral load, confirming that tuft cells act as a critical bottleneck for norovirus infection that, when expanded, enhances viral replication.

Beyond norovirus, tuft cells also respond to other viral infections, particularly enteroviruses. Recent studies have revealed important insights into the interactions between enteroviruses and tuft cells. Lyu et al. (47) demonstrated that various enteroviruses, including enterovirus 71 (EV71), coxsackievirus (CV) A16, CVB3, and CVB4, can upregulate IL-25 expression and induce tuft cell expansion in the intestinal tissues of mice. Their research uncovered a critical role for folate metabolism in supporting IL-25-induced tuft cell expansion during enteroviral infections. They found that folate was significantly enriched in intestinal tissues following both EV71 infection and recombinant murine IL-25 (rmIL-25) protein stimulation compared to control groups. Further investigation revealed that folate-deficient mice exhibited impaired tuft cell expansion in response to both EV71 infection and rmIL-25 stimulation, while folate supplementation enhanced tuft cell expansion in these models.

Building on these findings, Chen et al. (48) discovered that intestinal tuft cells can develop trained immunity during infancy, which enhances host defense against enteroviral infections later in life. This innovative concept reveals that tuft cells, despite being epithelial cells with a relatively short lifespan, can acquire immunological memory that strengthens subsequent antiviral responses. This trained immunity mechanism is associated with IL-25 pre-treatment, which enables tuft cells to respond more rapidly and strongly to viral challenges.

A recent transcriptomic study demonstrated that rotavirus (RV) infection affects intestinal tuft cells (49). The researchers found that, although RV infection did not significantly alter the number of tuft cells, it was capable of infecting approximately 5% of them, identifying tuft cells as a target for RV infection. Following infection, tuft cells primarily characterized by increased expression of chemosensory and immune response genes. Specifically, RV-infected tuft cells upregulated the expression of various interferon-stimulated genes (ISGs) and chemosensory-related genes, such as Plcb2 and Plcg2. Additionally, RV infection modified the immune functional characteristics of tuft cells; unlike the response to parasitic infection, RV infection decreased the expression of the antiparasitic cytokine IL-25. Furthermore, infected tuft cells enhanced luminal sampling in early tuft cells through increased uptake mechanisms, thereby improving their ability to monitor and defend against infection. (**Figure 1**)

In summary, tuft cells serve as rare but functional “sentinel” cells in the intestine, playing a crucial role in maintaining intestinal homeostasis and protecting against infections. These cells possess a variety of receptors capable of sensing diverse bacteria and viruses, enabling them to mount corresponding immune responses based on the specific microbial components they recognize, such as metabolites and viral proteins. In bacterial infections, tuft cells can recognize bacterial metabolites like N-C11-G through specific receptors such as Vmn2r26, triggering distinct immune pathways independent of the canonical ILC2-IL-13 axis. For viral infections, tuft cells demonstrate complex biology that can both facilitate viral persistence (as seen with norovirus immune privilege) and contribute to antiviral defense through interferon responses, trained immunity, and enhanced surveillance mechanisms. The newly discovered roles of folate metabolism in supporting IL-25-induced tuft cell expansion and the capacity of tuft cells to develop trained immunity further highlight the sophisticated functions of these cells in antiviral defense. This multifaceted nature highlights the context-dependent functions of tuft cells in viral infections, where they can serve as both viral reservoirs and critical components of antiviral immunity. (**Figure 1**).

## 3 The role of ILC2 in intestinal immunity

### 3.1 Overview of type 2 immune response in the gut

The intestine is the largest immune organ in the body, and the proper functioning of its immune system is essential for maintaining overall health. The intestinal immune system must not only defend against the invasion of foreign pathogens but also maintain tolerance to intestinal commensal microorganisms and food antigens (50). In the intestine, type 2 immune responses constitute a complex and sophisticated network that plays a crucial role in resisting parasitic infections and regulating allergic reactions, significantly contributing to intestinal homeostasis and influencing disease occurrence (51).

Within this network, ILC2, as key members of the innate immune system, enhance mucosal barrier function, increase the number of goblet cells and mucus secretion, and alter smooth muscle contractility through the secretion of specific cytokines. This process aids the body in clearing parasitic infections and inhibiting the occurrence of allergic reactions (52).

### 3.2 Developmental and phenotypic characterization of ILC2

ILC2 originate from common lymphoid progenitors in the bone marrow and gradually differentiate into mature ILC2 under the combined influence of the transcription factors GATA3, RORα, and TCF-1 (53). Additionally, the development of ILC2 is regulated by various cytokines (including IL-2, IL-7, and IL-33) and costimulatory molecules (such as ICOS and KLRG1) (52).

**Figure 2:**
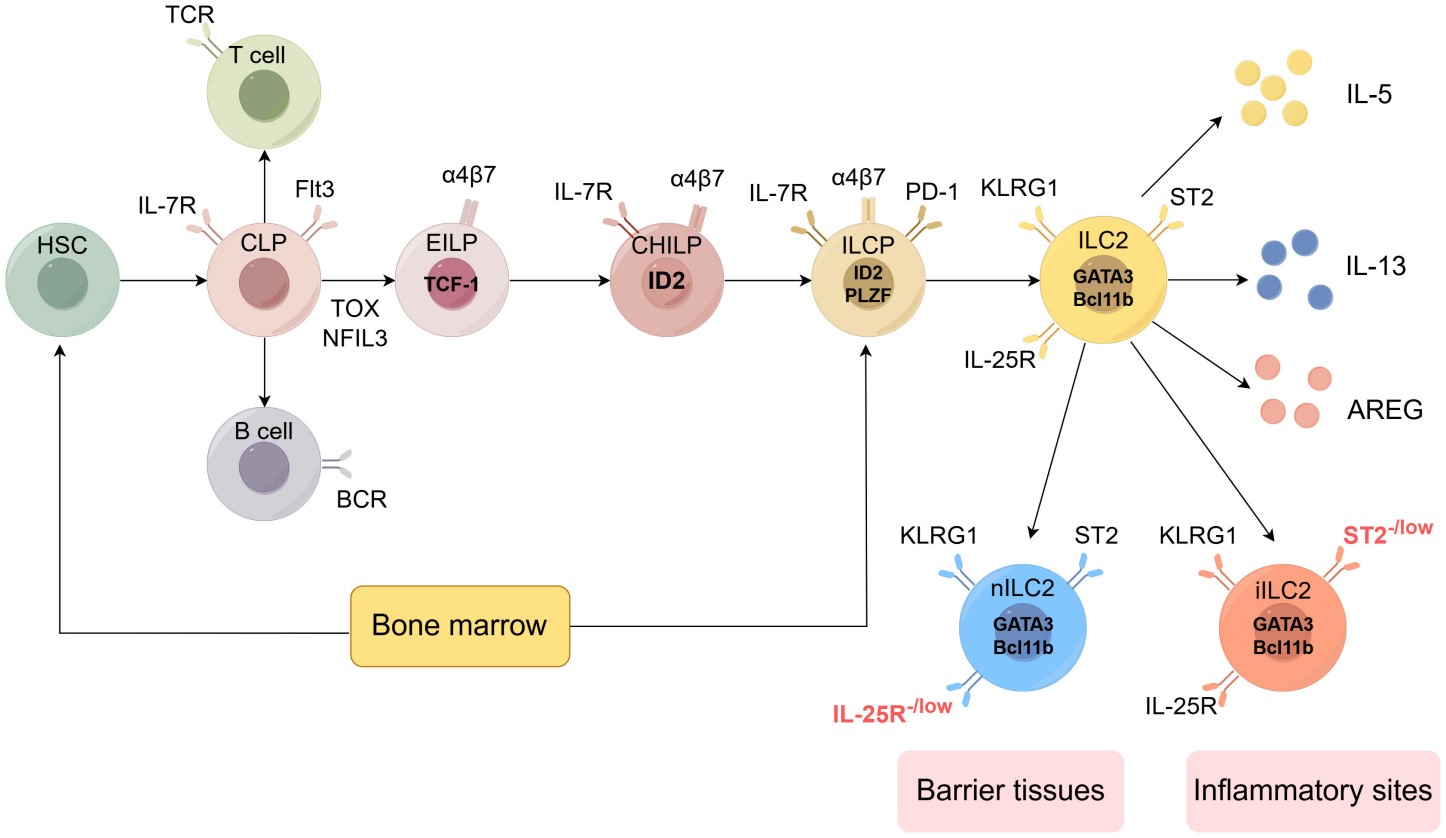
Development of the ILC2 (by Figdraw 2.0). Hematopoietic stem cells (HSCs) represent the origin of all blood cells, giving rise to common myeloid progenitors (CMPs) and common lymphoid progenitors (CLPs). CLPs further differentiate into all lymphocyte lineages. Early innate lymphoid cell progenitors (EILPs) develop into all ILC subsets, while common helper-like ILC progenitors (CHILPs) and ILC progenitors (ILCPs) retain multi-lineage potential. The figure shows key transcription factors (including TCF-1, ID2, PLZF, GATA3, Bcl11b), phenotypic markers (such as IL-7R, Flt3, α4β7, PD-1, KLRG1, ST2, IL-25R), and effector molecules (IL-5, IL-13, AREG) characterizing each developmental stage and mature ILC subset. ILC2 can be divided into two subtypes: nILC2 and iILC2. nILC2 resides in barrier tissues and expresses low levels of IL-25R, while iILC2 appears at inflammatory sites and is characterized by low levels of ST2 expression. Additional abbreviations: TCR, T cell receptor; BCR, B cell receptor; TOX, thymocyte selection-associated high mobility group box protein; NFIL3, nuclear factor interleukin 3-regulated. Adapted from Klose et al. Innate lymphoid cells control signaling circuits to regulate tissue-specific immunity. Cell Res. 2020;30(6):475-491. This adaptation is based on a figure licensed under a Creative Commons Attribution 4.0 International License (https://creativecommons.org/licenses/by/4.0/).

Mouse ILC2 are a subset of Lineage (Lin)- cells that lack the expression of mature hematopoietic cell lineage markers, including Ter119, Gr-1, CD11c, CD11b, and CD19. They predominantly express CD127 and Thy1, while also exhibiting a degree of heterogeneity through the expression of specific markers in various tissues. In the lungs, ILC2 typically express the IL-33 receptor, ST2, and are commonly characterized by the Lin-ST2+Thy1+CD25+ or Lin-CD45+CD127+ST2+ phenotypes. Recent evidence (54) indicates that ILC2 can be grouped into two distinct subsets: homeostatic or natural ILC2 (nILC2) and inflammatory ILC2(iILC2). nILC2 resides in barrier tissues such as the lung and adipose tissue, where it uniformly expresses ST2 (the IL-33 receptor) and is IL-17RB^-^ or IL-17RB^low^. nILC2 primarily responds to IL-33 stimulation and plays critical roles not only in immune protection but also in tissue repair and metabolic homeostasis. In the lungs, nILC2 is commonly characterized by the Lin-ST2+Thy1+CD25+ or Lin-CD45+CD127+ST2+ phenotypes. In contrast, iILC2 is not present in peripheral tissues in the steady state but can be rapidly elicited at many sites by helminth infection or IL-25 treatment. iILC2 uniformly expresses high levels of IL-17RB (the IL-25 receptor) and is ST2-. Therefore, IL-17RB is significant for identifying ILC2 that lack ST2 expression. Although iILC2 lacks ST2, it can become ST2+ nILC2-like cells following IL-25 administration or helminth infection, suggesting it can act as progenitors of nILC2-like cells. Conversely, in the small intestine, most ILC2 do not express ST2 but instead express the marker KLRG1. iILC2 also highly expresses the marker KLRG1. This tissue-specific marker profile is particularly relevant for understanding the tuft cell-ILC2 interaction, as intestinal tuft cells primarily activate ILC2 through IL-25 signaling rather than IL-33 (55). In humans, ILC2 express molecules such as CD161, CRTH2 (a prostaglandin D2 receptor), ST2, and IL-17RB, while not expressing lineage marker molecules (56). The expression of these characteristic surface markers aids in the identification and sorting of ILC2. (**Figure 2**)

### 3.3 Effect function of the ILC2

Intestinal ILC2 exhibits multifaceted effector functions that critically contribute to intestinal homeostasis and tissue repair.

ILC2 serves as a crucial line of defense in facilitating parasite clearance. As demonstrated by Moriyama et al., ILC2 is the primary producer of type 2 cytokines, particularly IL-5 and IL-13, which orchestrate critical protective mechanisms against helminth infections. IL-5 secreted by intestinal ILC2 directly drives the recruitment and activation of eosinophils in the lamina propria, while their IL-13 production stimulates goblet cell hyperplasia, enhances mucus secretion, and promotes intestinal epithelial turnover. These effector functions collectively contribute to the expulsion of mouse gastrointestinal helminths such as *Nippostrongylus brasiliensis*, as evidenced by the reduced worm burdens observed in experimental models with enhanced ILC2 activity (57).

In addition, Schneider et al. demonstrated that intestinal ILC2 responds to succinate, a metabolite produced by mutualistic colonizing protists, activating a tuft cell-ILC2 circuit. This activation leads to adaptive remodeling of the small intestine that not only increases surface area for absorption but also prevents subsequent helminth infections through a phenomenon known as concomitant immunity. The constitutive expression of IL-25 receptor (IL-17RB) on small intestinal ILC2, which is negatively regulated by A20 (Tnfaip3), underscores its specialized role in maintaining intestinal adaptability and immune surveillance (10).

Intestinal ILC2 also contributes significantly to tissue repair and maintenance of barrier integrity. Through the production of growth factors and cytokines, ILC2 promotes epithelial cell proliferation and restores intestinal barrier function following damage caused by inflammation. As demonstrated by Monticelli et al., intestinal ILC2 predominantly expresses amphiregulin (AREG) in response to IL-33 stimulation, which acts through the epidermal growth factor receptor (EGFR) signaling pathway to mediate epithelial repair. In the dextran sodium sulfate (DSS)-induced colitis mouse model, the IL-33-ILC2-AREG axis plays a critical role in ameliorating intestinal tissue damage and inflammation. Mechanistically, AREG promotes goblet cell hyperplasia, mucin production, and enhances tight junction protein expression such as Claudin-1, collectively strengthening the epithelial barrier. This reparative function is particularly important in the intestine, where continuous exposure to microorganisms and dietary antigens can potentially compromise epithelial integrity (58).

## 4 The tuft cell-ILC2 circuit: a critical mechanism in intestinal immunity

The tuft cell-ILC2 circuit represents a crucial signaling circuit that orchestrates type 2 immune responses in the intestinal epithelium. Tuft cells, a specialized chemosensory epithelial cell population, function as sentinels that detect luminal signals and initiate immune responses against parasitic infections through their communication with ILC2 (4, 17).

This interaction begins when tuft cells sense parasites or their metabolites through specialized receptors, including taste receptors such as TAS2R (26) and the succinate receptor Sucnr1 (36). Upon activation, tuft cells rapidly increase in number, expanding more than 15-fold within seven days of infection, and secrete the IL-25. IL-25 subsequently binds to IL-17RB receptors on ILC2, triggering their activation and expansion, as well as the production of type 2 cytokines, particularly IL-13. IL-13 completes the feedback circuit by acting on intestinal stem cells to promote the differentiation of more tuft cells and goblet cells, thus amplifying the initial response. This was demonstrated by von Moltke et al., who utilized IL-13, IL-4, or IL-4Rα knockout mouse models to assess their respective impacts on intestinal tuft cell expansion (4). The results indicated that the knockout of IL-13 or IL-4Rα significantly inhibited tuft cell expansion induced by parasitic infection, whereas the knockout of IL-4 did not have a notable effect. The finding suggests that IL-13 secreted by ILC2 plays an irreplaceable role in inducing tuft cell amplification in the intestinal epithelium through its binding to IL-4Rα, while IL-4 is not essential in this process.

Although Th2 cell and ILC2 belong to adaptive immunity and innate immunity respectively, both can secrete IL-13. This raises the question of whether Th2 cell is involved in the regulation of the tuft cell-ILC2 circuit. To address this, Howitt et al. infected *Rag2* knockout mice (deficient in T and B cells) and *Rag2/Il2rg* double knockout mice (lacking T cells, B cells, and ILC2) with *Tritrichomonas muris*. They observed that in *Rag2* knockout mice, significant tuft cell hyperplasia still occurred in the intestinal epithelium after infection. However, in *Rag2/Il2rg* double knockout mice, there was no significant difference in tuft cell numbers compared to uninfected controls. This suggests that the ILC2-IL-13 axis can function independently within the tuft cell-ILC2 circuit, without relying on Th2 cell-mediated adaptive immunity (6).

Recent research has expanded our understanding of this circuit beyond IL-25 signaling. McGinty et al. revealed that tuft cells also produce leukotrienes, particularly LTC_4_, which act as rapid mediators of anti-helminth immunity, working in parallel with the IL-25-ILC2-IL-13 circuit (39). Additionally, Ndjim et al. showed that tuft cells release ACh into the intestinal lumen, further contributing to anti-helminth defense mechanisms (30), (**Figure 1**).

## 5 Tuft cell- ILC2 circuit and the relationship between intestinal microbes, diet, and intestinal morphology

The intestinal ecosystem represents a complex environment where host immunity, microbial communities, and dietary factors continuously interact. In this context, the tuft cell-ILC2 circuit serves as a critical regulatory circuit that coordinates intestinal homeostasis and adaptive remodeling by sensing metabolic signals.

### 5.1 Microbial metabolites and tuft cell-ILC2 circuit activation

The gut microbiota plays a pivotal role in modulating intestinal immunity through the production of various metabolites. Recent research has revealed that commensal eukaryotic organisms, particularly protozoa such as *Tritrichomonas muris* (*T. muris*), significantly influence the tuft cell-ILC2 circuit. Howitt et al. (6) demonstrated that mice colonized with *T. muris* exhibited substantially higher numbers of intestinal tuft cells compared to specific-pathogen-free mice. Additionally, transferring cecal contents from *T. muris*-colonized mice to uncolonized mice was sufficient to induce tuft cell hyperplasia, indicating that *T. muris* or its metabolic products can activate the tuft cell-ILC2 circuit.

Schneider et al. (10) further elucidated this mechanism by showing that gut microbes, including protozoa, can ferment dietary fibers to produce short-chain fatty acids (SCFAs) and other metabolites such as succinate and acetate. Interestingly, tuft cells highly express G protein-coupled receptors for short-chain fatty acids (*Ffar3*) and succinate (Sucnr1/GPR91), suggesting their specialized role in detecting these microbial-derived signals. Their study demonstrated that germ-free mice monocolonized with *Tritrichomonas* exhibited increased cecal acetate and succinate levels. When succinate was provided in drinking water, it activated tuft cell-ILC2 circuit through the succinate receptor Sucnr1 in a Trpm5-dependent manner.

The work of Coutry et al. (59) complements these findings by demonstrating how disruptions in the intestinal microbial ecosystem, even in the absence of parasites, can influence the tuft cell-ILC2 circuit. Their findings suggest that Paneth cells play a key role in maintaining a balanced microbiota composition to prevent inappropriate activation of tuft cell-ILC2 circuit. When Paneth cell function was compromised, the resulting dysbiosis led to increased abundance of bacteria from the *Bacteroidales* order, which have higher potential for succinate production. This elevated succinate activated tuft cells through the SUCNR1 receptor, triggering a type 2 immune response.

### 5.2 Dietary regulation of the tuft cell-ILC2 circuit

Diet composition significantly influences the gut microbiota and consequently modulates the tuft cell-ILC2 circuit activation. Schneider et al. (10) revealed how specific dietary components, particularly fiber content, affect the colonization of protozoa and subsequently influence the tuft cell-ILC2 circuit. Their study demonstrated that post-weaning dietary changes coincide with increased *Tritrichomonas* abundance in the cecum and subsequent activation of the tuft cell-ILC2 circuit. When mice were maintained exclusively on a milk diet after weaning, they showed reduced tuft cell hyperplasia compared to mice on standard chow. Further experiments with fiber-manipulated diets showed that *Tritrichomonas* colonization was heavily reduced in mice fed diets lacking fiber or containing only cellulose as the fiber source. In contrast, when the oligofructan inulin was the sole dietary fiber, *Tritrichomonas* colonization was robustly established. This suggests that specific fermentable fibers provide essential metabolic substrates that support protozoa colonization and subsequent tuft cell activation. The specific mechanism involves protozoa like *Tritrichomonas* utilizing dietary fibers to produce metabolites, particularly succinate, which then activates the tuft cell-ILC2 circuit. This establishes a direct link between diet composition, microbiota metabolism, and tuft cell-ILC2 circuit activation.

### 5.3 Intestinal remodeling through the tuft cell-ILC2 circuit

Schneider et al. (10) confirmed that activation of the tuft cell-ILC2 circuit leads to changes in intestinal morphology. Specifically, they observed that mice with continuously activated tuft cell-ILC2 circuit (through A20(Tnfaip3) deletion in ILC2) exhibited adaptive intestinal lengthening. This remodeling was associated with changes in epithelial cell composition, including increased frequencies of tuft cells and goblet cells. The intestinal lengthening was dependent on IL-25 and IL-4Rα signaling, confirming that these structural changes were directly related to circuit activation. Furthermore, these adaptive morphological changes persisted even after protozoa clearance, suggesting that intestinal remodeling also possesses “memory” similar to adaptive immune T/B cells.

Coutry et al. (59) further extended our understanding of the intestinal remodeling process by demonstrating a three-step mechanism of dysbiosis establishment. Their work showed that following the initial tuft cell activation by altered microbiota, type 2 cytokines (particularly IL-13) produced by activated immune cells could modulate Paneth cell function. Specifically, IL-13 suppressed the expression of antimicrobial peptides *regenerating islet-derived 3-beta* (*RegIIIβ*) and –*gamma* (*RegIIIγ*) in Paneth cells, which further exacerbated dysbiosis. This created a feedback circuit where dysbiosis activated tuft cells, leading to type 2 inflammation, which further impaired Paneth cell function and worsened dysbiosis. Importantly, this intestinal remodeling process was absent in mice lacking tuft cells (*Pou2f3^-/-^*) or IL-4 receptor signaling (*IL-4Rα^-/-^*), highlighting the essential role of the tuft cell-ILC2 circuit in mediating these changes.

In summary, symbiotic microorganisms, particularly eukaryotic symbionts such as the protozoan *T. muris*, can produce metabolites like succinate by metabolizing specific fibers from the host diet. These metabolites then act on receptors on the tuft cell surface to trigger the tuft cell-ILC2 circuit, leading to changes in intestinal morphology including increases in intestinal tuft cells and small intestinal length. This reveals the complex interrelationship between diet, gut microbiota, and intestinal homeostasis.

## 6 The role of the tuft cell-ILC2 circuit in intestinal diseases

### 6.1 The role of the tuft cell-ILC2 circuit in human intestinal diseases

Intestinal diseases, such as inflammatory bowel disease (IBD), are a group of chronic recurrent diseases that include ulcerative colitis (UC) and Crohn’s disease (CD). The pathogenesis of IBD is multifactorial, involving immune disorders, intestinal microbiota imbalances, genetic susceptibility, environmental factors, and many other aspects (60). A distinctive feature of inflammatory bowel disease (IBD) is the alternation between periods of exacerbation and remission of inflammation. The inflammatory phase is characterized by increased secretion of type 1 cytokines, such as IFN-γ and TNF-α, which disrupts the balance between type 1 and type 2 immunity. Recent investigations have demonstrated that IL-25 levels are significantly reduced in both the serum and inflamed intestinal mucosa of patients with acute-onset IBD. Importantly, this reduction in IL-25 has also been observed in non-inflammatory tissue and serum from patients with quiescent UC and CD (61). These findings suggest that tuft cells, as the primary source of IL-25, may play a protective role in intestinal inflammation in humans.

Further analyses of human intestinal biopsy samples have shown alterations in tuft cell numbers and morphology in IBD patients compared to healthy controls. The decreased IL-25 levels correlate with reduced tuft cell populations in the intestinal epithelium of these patients, supporting the hypothesis that impaired tuft cell-ILC2 circuit signaling contributes to disease pathogenesis (62).

### 6.2 The role of the tuft cell-ILC2 circuit in mouse models of intestinal inflammation

Mouse models have revealed the protective function of the tuft cell-ILC2 circuit against intestinal inflammation. In a dextran sulfate sodium (DSS)-induced colitis mouse model, administration of recombinant IL-25 significantly reduced clinical symptoms of colitis, including weight loss and colon ulceration, and prolonged the survival period of the mice (63). This protective effect was associated with a shift from type 1 to type 2 immune responses in the intestinal mucosa.

Recent research has revealed that berberine, a plant alkaloid widely used in traditional Chinese medicine for treating diarrhea, can ameliorate dextran sulfate sodium (DSS)-induced colitis through a tuft cell-dependent mechanism. Yang et al. demonstrated that berberine promoted the expansion of tuft cells and stimulated IL-25 secretion in the colonic epithelium, subsequently activating ILC2 and Th2 cell. The therapeutic effects of berberine were significantly diminished in *Pou2f3* knockout mice (which lack tuft cells), confirming the essential role of tuft cells in mediating berberine’s anti-inflammatory properties. Further investigation revealed that berberine exerts its effects by TAS2Rs on tuft cells, as the beneficial effects were partially abolished by U73122, a bitter taste receptor inhibitor (64). This study provides compelling evidence that pharmacological targeting of tuft cells may represent a promising therapeutic approach for intestinal inflammation.

## 7 Discussion

Recent mechanistic discoveries have revolutionized our understanding of the tuft cell-ILC2 circuit. The identification of specific receptors and signaling pathways, notably Sucnr1 for succinate detection and TAS2R for sensing bitter compounds from helminth parasites, has provided molecular insights into how tuft cells detect diverse pathogens. These findings establish tuft cells as specialized sentinels at the intestinal epithelium. However, the diversity of pathogens recognized by tuft cells, including bacteria and viruses that do not produce succinate or bitter compounds, strongly suggests the existence of additional, undiscovered sensing mechanisms.

The dual functionality of tuft cells—as both immune sensors and direct effectors—represents a particularly significant breakthrough. The discoveries by Billipp et al. and Ndjim et al. that tuft cell-derived Ach directly inhibits helminth reproduction and promotes epithelial chloride secretion have fundamentally expanded our understanding of anti-helminth defense. This direct effector function operates in parallel with the canonical IL-25-mediated activation of type 2 immunity, revealing a sophisticated two-pronged defense strategy. Evolutionarily, this arrangement appears advantageous, allowing for immediate local responses through Ach while simultaneously initiating longer-term adaptive changes through ILC2 activation.

It is interesting that the tuft cell-ILC2 circuit functions beyond helminth defense. The finding that this circuit responds to dietary fiber and microbiota-derived metabolites to drive intestinal remodeling suggests an evolutionary adaptation to optimize nutrient absorption in response to food availability. Similarly, the unexpected role of this circuit in viral infections—sometimes beneficial (as with enteroviruses) and sometimes detrimental (as with norovirus persistence)—indicates context-dependent functions that go well beyond the canonical type 2 immune response.

However, the current research on the tuft cell-ILC2 circuit is still in its infancy, and there are still many problems to be further explored. Firstly, the question of whether the immunoregulatory capacity of the tuft cell-ILC2 circuit is confined to type 2 immunity or can be extended to other types of immune responses warrants further investigation. Secondly, it is crucial to identify the ligands recognized by tuft cells, the receptors involved in their activation, and the corresponding signaling pathways. While tuft cells are known for their unique chemoreceptive abilities and responses to bitter substances, the mechanisms by which they integrate signals from diverse pathogen-related molecular patterns remain to be elucidated. Furthermore, in addition to secreting IL-25, it is important to consider whether activated tuft cells can also regulate the local immune microenvironment through alternative mechanisms. Additionally, it is necessary to explore whether ILC2’s epigenetic modifications and metabolic reprogramming are altered following stimulation by factors such as IL-25, and whether the tuft cell-ILC2 circuit interacts with other immune cells, such as macrophages and dendritic cells, in the intestinal lamina propria. Investigating the potential cross-regulation among innate immune cells will enhance our understanding of the dynamic balance mechanisms governing intestinal mucosal immunity. Moreover, the dynamic changes in the number, phenotype, and function of tuft cells and ILC2 under disease conditions, as well as the underlying mechanisms, require comprehensive analysis. For instance, in the context of chronic intestinal inflammation, could reduce tuft cell-ILC2 circuit activity serve as a driver of disease? Is this activity negatively regulated by other immune cells? Addressing these questions may facilitate the identification of new disease markers and therapeutic targets, ultimately leading to significant advancements in the prevention and treatment of intestinal diseases.

Currently, data from clinical samples and studies involving human subjects are relatively limited. Given the significant differences between mice and humans in terms of anatomy, immune systems, and commensal microorganisms, it is essential to enhance human studies in the future. This can be achieved by utilizing endoscopic biopsy specimens, surgical resection tissues, and patient clinical data to conduct a comprehensive analysis of the characteristics of ILC2 circuits in humans and their correlation with disease prognosis. Such efforts not only deepen our understanding of the disease but also facilitate the translation of basic research findings into clinical applications.
