# Correction: Regulation of the tuft cell-ILC2 circuit in intestinal mucosal immunity

**DOI:** 10.3389/fimmu.2025.1636022

**Published:** 2025-06-12

**Authors:** Kaiyu Shang, Xinxin Qi, Tingting Tian, Huidong Shi, Yuejie Zhu, Fengbo Zhang

**Affiliations:** ^1^ Department of Clinical Laboratory, The First Affiliated Hospital of Xinjiang Medical University, Urumqi, Xinjiang, China; ^2^ Reproductive Medicine Center, The First Affiliated Hospital of Xinjiang Medical University, Urumqi, Xinjiang, China

**Keywords:** tuft cells, mucosal immunity, intestinal homeostasis, inflammatory bowel disease, tuft cell-ILC2 circuit

In the published article, there was an error in the legend for **Figure 1** as published. Text that should have appeared in the figure caption, was erroneously included in the body text of the article. The corrected legend appears below.

**Figure 1:**
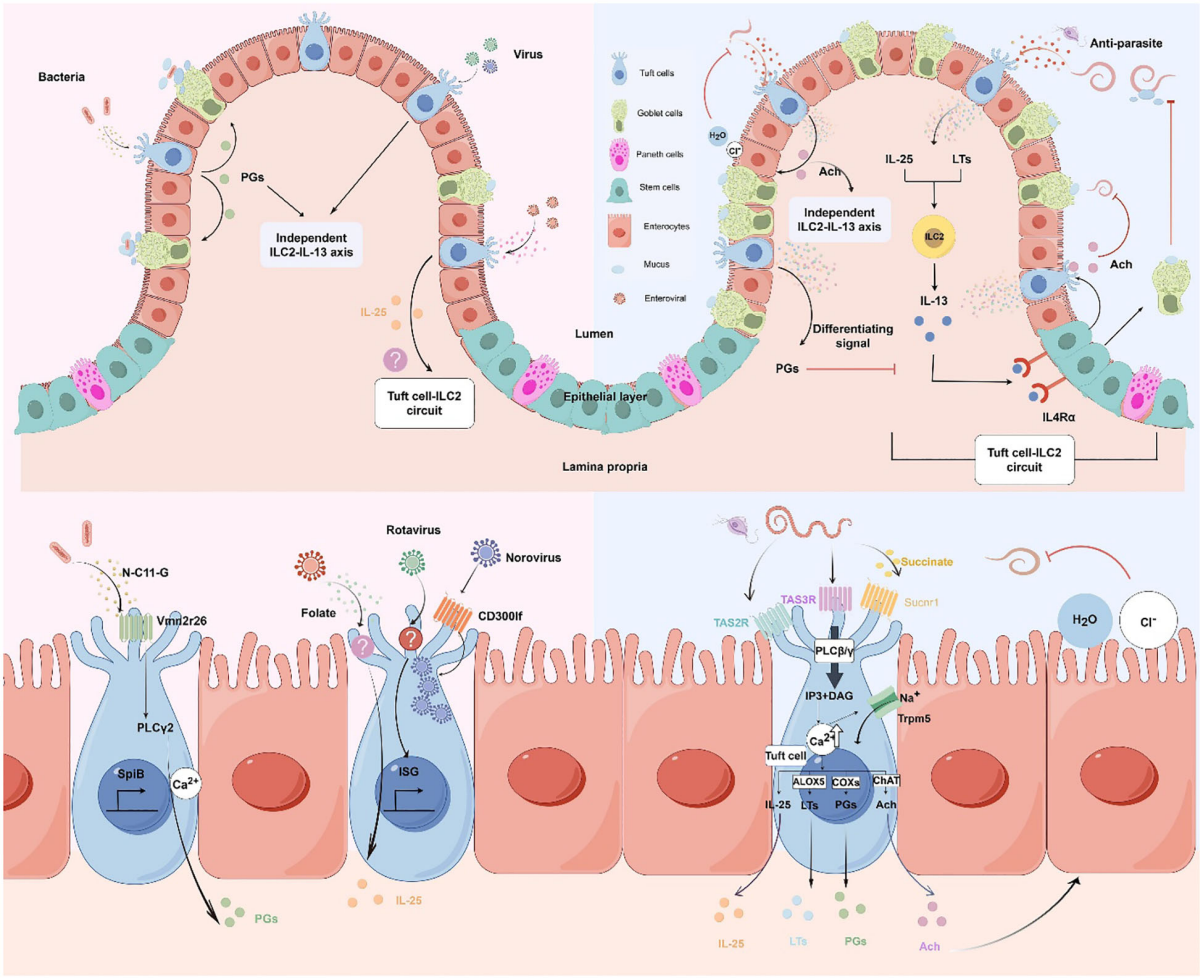
Molecular and cellular signaling of intestinal tuft cells in response to infections. (by Figdraw 2.0). Left panels: Tuft cell detection of bacterial and viral infections. Upper left: Overview of tuft cells recognizing bacterial and viral pathogens. Lower left: Magnified view of molecular mechanisms. Bacterial metabolite N-C11-G from *Shigella* is recognized by Vmn2r26 GPCR expressed on tuft cells, activating PLCγ2-Ca^2+^ signaling pathway. This leads to PGs production and SpiB-induced tuft cell hyperplasia. Norovirus binds to CD300lf receptors on tuft cells, leading to viral replication. Rotavirus triggers tuft cell activation through an unknown receptor, resulting in ISG expression that contributes to antiviral defense. Enteroviruses upregulate IL-25 expression and induce tuft cell expansion through folate metabolism, but it is uncertain whether it activates type 2 immune responses. Right panels: Tuft cell-mediated response to parasitic infections. Upper right: Overview of tuft cells in anti-parasitic immunity. Tuft cells detect parasite metabolites and produce IL-25 and LTs, which activate ILC2 to secrete IL-13. IL-13 binds to IL4Rα on intestinal stem cells, promoting differentiation into tuft cells and goblet cells. Goblet cells secrete mucus to aid in parasite clearance, establishing the tuft cell-ILC2 circuit. However, PGs counteract IL-13 effects. Moreover, tuft cells secrete Ach which can directly inhibit helminth reproduction or act on adjacent epithelial cells, inducing chloride secretion followed by water secretion, thereby promoting worm expulsion. Lower right: Magnified view of molecular mechanisms. Succinate binds to Sucnr1, while TAS2R and TAS3R, all GPCRs, detect other parasite-derived metabolites. Receptor activation triggers phospholipase C (PLCβ/γ), converting PIP2 to IP3 and DAG, leading to increased intracellular Ca^2+^ and Trpm5 channel opening. This cascade results in IL-25 release, enhanced ChAT expression, and activation of eicosanoid pathway-related proteins (COX1, COX2, and ALOX5). Adapted from Seminars in Cell & Developmental Biology, Vol. 150-151, Bas J, Jay P, Gerbe F, Intestinal tuft cells: Sentinels, what else?, Pages 35-42, Copyright (2023), with permission from Elsevier.

In the published article, there was an error in the legend for **Figure 2** as published. Text that should have appeared in the figure caption, was erroneously included in the body text of the article. The corrected legend appears below.

**Figure 2:**
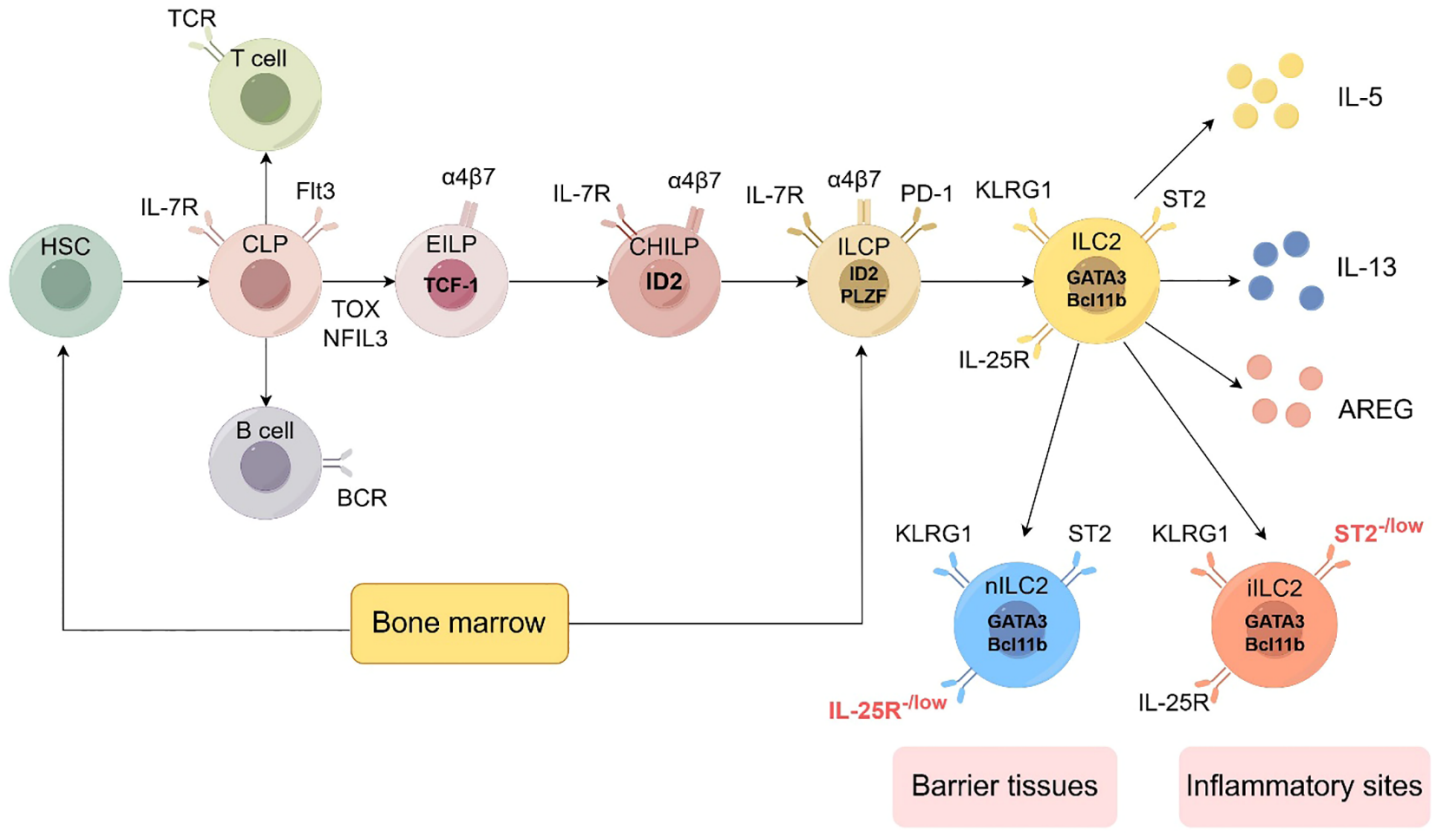
Development of the ILC2 (by Figdraw 2.0). Hematopoietic stem cells (HSCs) represent the origin of all blood cells, giving rise to common myeloid progenitors (CMPs) and common lymphoid progenitors (CLPs). CLPs further differentiate into all lymphocyte lineages. Early innate lymphoid cell progenitors (EILPs) develop into all ILC subsets, while common helper-like ILC progenitors (CHILPs) and ILC progenitors (ILCPs) retain multi-lineage potential. The figure shows key transcription factors (including TCF-1, ID2, PLZF, GATA3, Bcl11b), phenotypic markers (such as IL-7R, Flt3, α4β7, PD-1, KLRG1, ST2, IL-25R), and effector molecules (IL-5, IL-13, AREG) characterizing each developmental stage and mature ILC subset. ILC2 can be divided into two subtypes: nILC2 and iILC2. nILC2 resides in barrier tissues and expresses low levels of IL-25R, while iILC2 appears at inflammatory sites and is characterized by low levels of ST2 expression. Additional abbreviations: TCR, T cell receptor; BCR, B cell receptor; TOX, thymocyte selection-associated high mobility group box protein; NFIL3, nuclear factor interleukin 3-regulated. Adapted from Klose et al. Innate lymphoid cells control signaling circuits to regulate tissue-specific immunity. Cell Res. 2020;30(6):475-491. This adaptation is based on a figure licensed under a Creative Commons Attribution 4.0 International License (https://creativecommons.org/licenses/by/4.0/).

In the published article, there was an error. Text to be used in the figure captions was erroneously included in the body text of the article.

A correction has been made to **2 Tuft cells: sentinels of the intestinal epithelium**, *2.3.2 Role of tuft cells in bacterial and viral infections*, Paragraphs 9 and 10. These paragraphs should be removed from the section.

A correction has also been made to **3 The role of ILC2 in intestinal immunity**, *3.2 Developmental and phenotypic characterization of ILC2*, Paragraph 3. This paragraph should be removed from the section.

The original article has been updated.

